# Artificial Intelligence-Based Atrial Fibrillation Recognition Method for Motion Artifact-Contaminated Electrocardiogram Signals Preprocessed by Adaptive Filtering Algorithm

**DOI:** 10.3390/s24123789

**Published:** 2024-06-11

**Authors:** Huanqian Zhang, Hantao Zhao, Zhang Guo

**Affiliations:** 1 Shanghai Institute of Microsystem and Information Technology, Chinese Academy of Sciences, Shanghai 200050, China; 2 Key Laboratory of Intelligent Perception, and Image Understanding of Education Ministry of China, School of Artificial Intelligence, Xidian University, Xi’an 710071, China; 21171213924@stu.xidian.edu.cn; 3 Academy of Advanced Interdisciplinary Research, Xidian University, Xi’an 710071, China

**Keywords:** atrial fibrillation, motion artifact, electrocardiogram, adaptive filtering, artificial intelligence

## Abstract

Atrial fibrillation (AF) is a common arrhythmia, and out-of-hospital, wearable, long-term electrocardiogram (ECG) monitoring can help with the early detection of AF. The presence of a motion artifact (MA) in ECG can significantly affect the characteristics of the ECG signal and hinder early detection of AF. Studies have shown that (a) using reference signals with a strong correlation with MAs in adaptive filtering (ADF) can eliminate MAs from the ECG, and (b) artificial intelligence (AI) algorithms can recognize AF when there is no presence of MAs. However, no literature has been reported on whether ADF can improve the accuracy of AI for recognizing AF in the presence of MAs. Therefore, this paper investigates the accuracy of AI recognition for AF when ECGs are artificially introduced with MAs and processed by ADF. In this study, 13 types of MA signals with different signal-to-noise ratios ranging from +8 dB to −16 dB were artificially added to the AF ECG dataset. Firstly, the accuracy of AF recognition using AI was obtained for a signal with MAs. Secondly, after removing the MAs by ADF, the signal was further identified using AI to obtain the accuracy of the AF recognition. We found that after undergoing ADF, the accuracy of AI recognition for AF improved under all MA intensities, with a maximum improvement of 60%.

## 1. Introduction

Atrial fibrillation (AF), commonly referred to as AF, is a prevalent arrhythmia, and its timely diagnosis is crucial for patient management and treatment. The occurrence of AF is associated with various physiological and pathological conditions, including exercise. Studies have shown that high-intensity, short-duration exercise can lead to overactivation of the sympathetic nervous system, while prolonged endurance exercise may increase parasympathetic tone. These physiological responses can shorten the action potential duration and effective refractory period of atrial myocytes, increase the autoactivation of atrial myocytes, and thereby increase the risk of AF [1,2]. Unfortunately, electrocardiogram (ECG) monitoring in non-static environments, especially during exercise, is often disturbed by motion artifacts (MAs). These artifacts are primarily caused by relative movement between the electrodes and the skin. Nearly all non-rest activities generate electromagnetic noise, which is particularly common in routine ECG monitoring [3]. The presence of these artifacts not only affects the quality of the ECG signal but also reduces the accuracy of QRS wave recognition [4]. In ECG, AF is typically characterized by irregularity of QRS waves and the absence of P waves [5]. Therefore, the presence of MAs poses a significant barrier to the accurate identification of AF.

**Figure 1:**
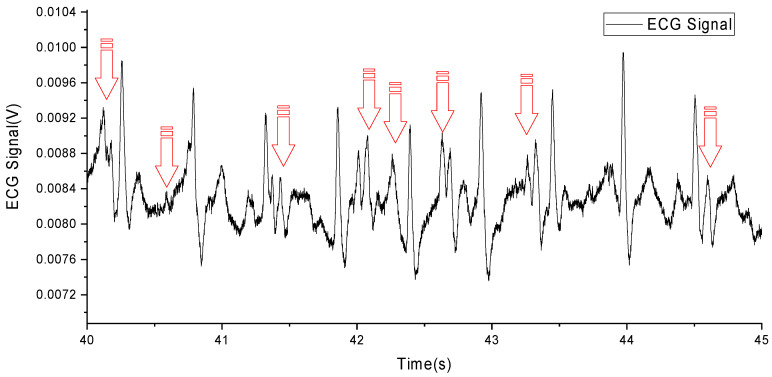
An ECG with MA caused by running.

However, it remains challenging to remove the disturbance of MAs from an ECG signal. Due to the overlap of MAs and the components of the ECG signal in frequency [6], as shown in Figure 1 (MAs are marked with red arrows) and Table 1, traditional filtering methods are difficult to effectively remove MAs.

To address the issue of MA, it has been previously proposed to sand the skin to remove the stratum corneum [7]. However, damaged skin will grow back during long-term monitoring, and the skin may also become infected due to damage, so this method is not suitable for early screening of AF. Another approach to eliminate MA is through signal processing, which commonly involves adaptive filtering algorithms and blind source separation algorithms [8].

Blind source separation (BSS) algorithms can extract independent source signals from measured signals based on statistically independent relationships between the source signals [9]. Due to the origin of biometric signals from the activities of different organs and tissues in the human body, they are statistically independent and orthogonal in multidimensional space. Therefore, BSS methods can be used to extract different independent signals, including MA signals that are different from the source of ECG signals. However, this method requires a large amount of computation and requires multi-channel ECG signals for correlation analysis, which is not suitable for long-term early screening applications of AF.

Adaptive filtering technology combined with a reference signal with a strong correlation to MAs can effectively eliminate MAs and improve the accuracy and reliability of the ECG signal [10]. Adaptive filtering is a technique that can automatically adjust filter parameters based on the characteristics of the reference signal [11], and is widely used in biometric signal processing. It can be used to process ECG signals of the fetus [11], eliminate 50 Hz power line interference [11,12], eliminate electromyographic noise interference [13], and eliminate base-line shifts in ECG signals [12,13]. In addition, it has a low computational burden [14], high reliability, and can handle single-channel ECG signals [15], making it very suitable for long-term early screening applications of AF.

In recent years, deep learning models have played a pivotal role in the detection and diagnosis of AF [1,2,3,4,5]. To enhance the efficiency and accuracy of AF recognition, researchers have proposed ECG signal classification models based on convolutional neural networks (CNN) [16] and improved deep learning models that combine CNN with long short-term memory (LSTM) networks [17]. These models effectively extract and deeply mine features from ECG signals to achieve AF recognition [18]. Additionally, there have been studies presenting intelligent diagnosis methods based on one-dimensional convolutional neural networks [19] and AF detection methods combining the Hilbert–Huang transform with deep convolutional neural networks [20]. These methods have demonstrated a reasonable performance and proved its strong generalization ability and robustness. However, MAs still significantly undermine the performance of deep learning models when processing real-world data [21]. Recent study has focused on denoising methods based on neural network autoencoders, achieving encouraging results [22]. Nevertheless, the test sets used in these studies are relatively small and do not include real-time dynamic ECG signals, limiting their widespread application in practical scenarios.

In summary, MAs can affect the accuracy of AF recognition, adaptive filtering can effectively eliminate MAs, and deep learning algorithms can effectively identify AF. Therefore, it is necessary to study whether the preprocessing of adaptive filtering algorithm for ECG signals containing MAs can help deep learning further improve the accuracy of AF recognition. However, due to the fact that existing databases of AF ECG signals generally do not contain MAs, there is no reference signal required for adaptive filtering that is correlated with MAs. There has not been study available reporting on the accuracy of AF recognition for ECG signals containing MA.

Given the lack of strong correlated reference signals in publicly available ECG databases provided by authoritative institutions, research worldwide had adopted methods of self-developed reference signal detection equipment to address this issue. Reference signals were mainly divided into three categories: electrode skin contact impedance, skin deformation, and three-axis acceleration, and Table 2 provides their classification. We developed a reference signal detection device that can detect skin stretch deformation synchronized with the ECG signal.

Therefore, in this study, our research can be separated as three steps:

1. Proposing a method of artificially adding MA to AF ECG signals.
2. Proposing preprocessing of the aforementioned ECG signals containing MA using adaptive filtering algorithms before identifying AF by AI algorithms.
3. The AI algorithm that combines knowledge guidance and attention has been applied in AF identification.

In summary, this paper innovatively combines an adaptive filter algorithm with AI techniques, proposing a novel multi-stage algorithm to identify atrial fibrillation in ECG signals affected by motion artifacts.

The second section of this paper will introduce the basic ECG features for recognizing AF, introduce the detection methods for skin stretch deformation signals strongly related to MAs, introduce the algorithm for adding MAs to the ECG, introduce the adaptive filtering algorithm, and introduce the neural network algorithm for AF recognition. The third section of this paper will introduce the experimental methods and results, and the fourth section will discuss the final experimental results (accuracy of AF recognition). The fifth section will summarize this paper.

## 2. Method

### 2.1. Basic ECG Characteristics of AF

**Figure 2:**
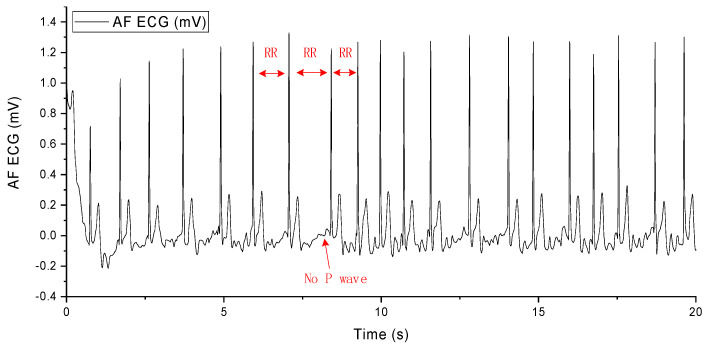
AF ECG signal.

The occurrence of AF arises from abnormal electrical impulses suddenly originating from the atria. These impulses disrupt the normal rhythm of the heart and overshadow its natural pacemaker activity. AF causes irregular and abnormally rapid contractions of myocardial cells, leading to alterations in the heart’s rhythm and resulting in an irregular heartbeat, palpitations, dizziness, shortness of breath, and fatigue. Diagnosis of AF from an ECG primarily involves observing the following three characteristics in the electrocardiographic signal: unequal RR intervals, the disappearance of the P wave, and the appearance of the F wave, as shown in Figure 2. When these three features are present in the ECG, a diagnosis of AF can be made [50].

### 2.2. Detection of Skin Stretching Signals

**Figure 3:**
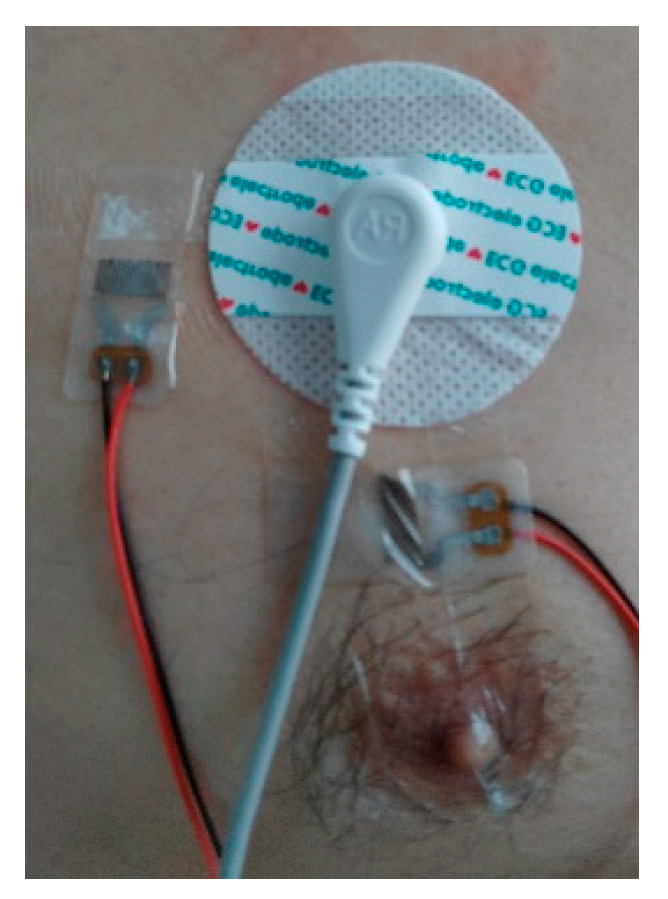
Installation of stretch sensors.

**Figure 4:**
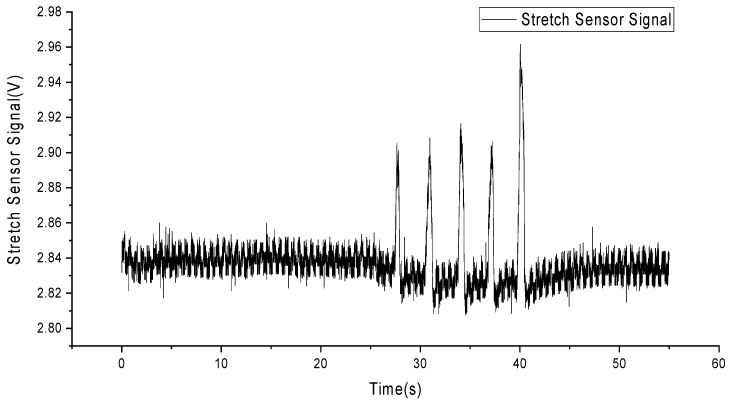
Skin stretching deformation signals.

To detect skin stretching signals, we employed flexible and stretchable sensors made from liquid metal particles [51]. These sensors were attached to the skin surface a few millimeters away from the electrodes, with one sensor placed horizontally and the other vertically to the ground plane, as shown in Figure 3. The circular patch in the top right corner represents a traditional electrocardiogram electrode, while two elongated stretch sensors are placed to the left and below it. The sensor signals were processed through an amplifier, converted to digital signals by an analog-to-digital converter, and then saved as data files on a computer. Figure 4 shows the deformation signals captured by the sensors when the skin was stretched manually, with a total of five stretches performed.

### 2.3. Algorithm for Adding MA to ECG

In our study, we utilized the AF dataset from the PhysioNet Challenge 2017 database, which contains a large collection of ECG signal data from patients with AF. To better understand and evaluate the performance of deep learning models in processing ECG signals contaminated with MA, we artificially added motion artifact signals with signal-to-noise ratios (SNRs) ranging from +8 dB to −16 dB.

First, we conducted all experiments using authentic ECG data from the PhysioNet Challenge 2017 database [52]. This dataset comprises 8528 de-identified (aimed at ensuring data privacy and preventing identification of specific individuals) ECG records, including 738 from AF patients and 7790 from a non-AF control group. The durations of these records range from 9 s to just over 60 s, with a sampling frequency of 300 Hz. The richness of this dataset makes it an ideal choice for evaluating models for arrhythmia recognition and classification.

Next, we added calibrated amounts of noise from various motion artifact noise records to the authentic ECG data (from the PhysioNet Challenge 2017 database) to assess the noise tolerance and performance improvement of our approach. Since the ECG signals in the clean records were sampled at 300 Hz, and the motion artifact noise records were sampled at 1000 Hz, resulting in a frequency difference exceeding 10%, we down sampled the motion artifact noise records to generate new 300 Hz sampled noise records.

In the testing phase, we characterized the noise level using the SNR. SNR is a metric that describes the relative strength of a signal compared to the background noise, typically measured in decibels (dB). It is a crucial indicator for assessing signal quality and is particularly important in fields such as communication systems and signal processing. The SNR is calculated using the following formula:

(1) $$SNR=10\log\left(S/N\right)$$

where *S* represents the power of the signal, and *N* represents the power of the noise.

Each clean ECG signal (clean signal) was paired with a noise signal (noise signal). From the noise record, the calibrated amount of noise ‘a∗noise signal’ was added to the clean signal using the following formula:

(2) output_signal=clean signal+a∗noise signal

Where the ‘*output signal*’ represents the motion artifact ECG signal with MA, and ‘*a*’ is the gain. The gain ‘*a*’ is specified by the SNR and is influenced by the signal’s power *S* and the noise’s power *N*:

(3) $$a=\sqrt{\frac{S}{N}\times 10^{-\frac{SNR}{10}}}$$

This scaling factor adjusts the amplitude of the noise signal so that the power of the noise signal added to the original signal equals the target noise power, maintaining a specific *SNR*. This allows us to simulate ECG signals in real-world scenarios where patients are performing daily activities. This is crucial for evaluating and improving the performance of deep learning models in processing ECG signals contaminated with MA.

### 2.4. Adaptive Filter Algorithm

**Figure 5:**
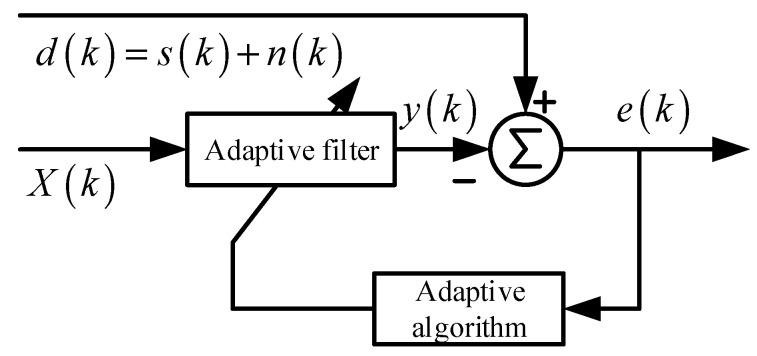
The block diagram of noise cancelling AF.

The adaptive noise cancellation algorithm requires the output signal of a primary sensor as the desired signal $d\left(k\right)$ and the output signal of another sensor as the reference signal $x\left(k\right)$. The desired signal consists of an ideal, undistorted signal $s\left(k\right)$ and a noise signal $n\left(k\right)$. It is assumed that there is no correlation between the ideal undistorted signal $s\left(k\right)$ and the noise signal $n\left(k\right)$, while the reference signal $x\left(k\right)$ is correlated with the noise signal $n\left(k\right)$, as shown in Figure 5.

Widrow [11] demonstrated that minimizing the mean square error (MSE)—the mathematical expectation $E\left[e\left(k\right)\right]$ of $e\left(k\right)$—results in the undistorted, ideal signal with the noise removed.

Assuming that $s\left(k\right)$, $n\left(k\right)$, $x\left(k\right)$, and $y\left(k\right)$ are statistically stationary signals with zero mean, the output of the filter is given by:

(4) $$e\left(k\right)=s\left(k\right)+n\left(k\right)-y\left(k\right)$$

Taking the mean square of both sides of the equation above, and given that the signal $s\left(k\right)$ is uncorrelated with both $n\left(k\right)$ and $y\left(k\right)$, we obtain the following expression:

(5) $$E\left[e^{2}\left(k\right)\right]=E\left[s^{2}\left(k\right)\right]+E\left[{\left(n\left(k\right)-y\left(k\right)\right)}^{2}\right]+2E\left[s\left(k\right)\left(n\left(k\right)-y\left(k\right)\right)\right]\;=E\left[s^{2}\left(k\right)\right]+E\left[{\left(n\left(k\right)-y\left(k\right)\right)}^{2}\right]$$

By employing an appropriate filter algorithm, we can minimize $E\left[e^{2}\left(k\right)\right]$. At this point, the second term on the right-hand side of the equation is also minimized.

(6) $$minE\left[e^{2}\left(k\right)\right]=E\left[s^{2}\left(k\right)\right]+minE\left[{\left(n\left(k\right)-y\left(k\right)\right)}^{2}\right]$$

Therefore, the output $y\left(k\right)$ of the filter serves as the least squares estimate of the noise $n\left(k\right)$. Similarly, since $e\left(k\right)-s\left(k\right)=n\left(k\right)-y\left(k\right)$, when $E\left[{\left(n\left(k\right)-y\left(k\right)\right)}^{2}\right]$ is minimized, $E\left[{\left(e\left(k\right)-s\left(k\right)\right)}^{2}\right]$ is also minimized. Consequently, minimizing the mean square error results in the error signal *e*(*k*) being the best least squares estimate of the original undistorted signal *s*(*k*). When $E\left[{\left(n\left(k\right)-y\left(k\right)\right)}^{2}\right]=0$, we have $E\left[e^{2}\left(k\right)\right]=E\left[s^{2}\left(k\right)\right]$, indicating that at this point, $n\left(k\right)=y\left(k\right)$ and $e\left(k\right)=s\left(k\right)$. Therefore, the error signal $e\left(k\right)$ represents the ideal, undistorted signal $s\left(k\right)$ free from noise.

In our study, we used the standard ADF structure, as shown in Figure 4. The least mean square (LMS) was used as adaptive algorithm. The equation of this ADF is as follows:

(7) $$\left\{\begin{array}{c} Y\left(k\right)=W\left(k\right)\cdot X\left(k\right)\\ \begin{array}{c} W\left(k+1\right)=W\left(k\right)+\mu \cdot e(k)\cdot X(k)\\ \begin{array}{c} \mu =\beta \cdot \left[0.5+exp\left(-\alpha \cdot \left|e(k)\right|\right)\right]\\ \begin{array}{c} \alpha =0.01\\ \beta =0.0005 \end{array} \end{array} \end{array} \end{array}\right.$$

### 2.5. Brief Overview of the Neural Network Algorithm for AF Recognition

Multi-layer knowledge-guided attention network: This is a deep learning-based approach that can predict cardiac diseases from ECG signals while providing intuitive interpretations.

Multi-layer feature extraction and fusion: This method utilizes convolutional neural networks and bi-directional long short-term memory networks to extract rhythm, morphology, and frequency features from ECG signals. These features are then fused through a multi-layer attention mechanism.

Knowledge-guided attention mechanism: This approach leverages knowledge from the ECG domain to guide the computation of attention, enabling the model to focus on signal portions relevant to diagnosis. This enhances the interpretability and accuracy of the model.

#### 2.5.1. Introduction to Neural Networks for AF Recognition

**Figure 6:**
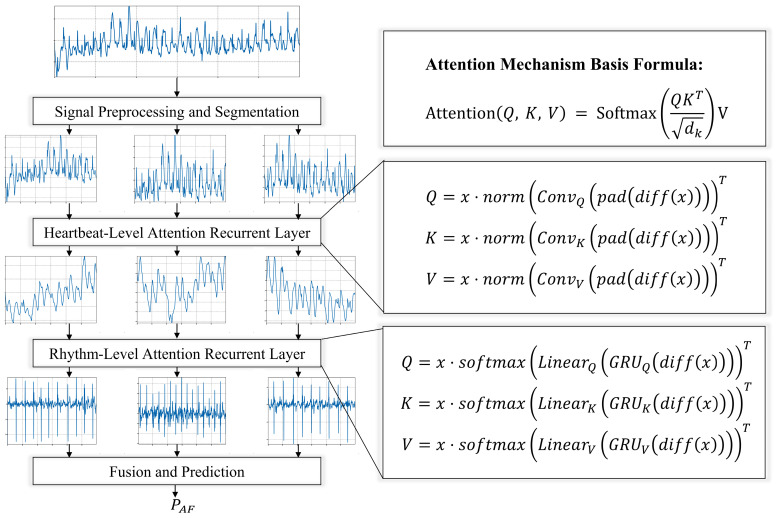
Atrial fibrillation recognition neural network structure diagram.

In our study, we introduce a neural network model for atrial fibrillation detection, which processes single-lead ECG signals and employs multi-level attention mechanisms, as depicted in Figure 6. The model initially segments the single-lead ECG signals into multiple fragments, and then applies a beat-level convolutional attention layer and a rhythm-level recurrent attention layer to each fragment to capture beat-level and rhythm-level attention information. Subsequently, a fusion and prediction layer perform dimension transformation and integrates hierarchical information to predict the category probabilities of heartbeat signals. The incorporation of a knowledge-guided attention mechanism further enhances the model’s performance and interpretability, specifically learning attention weights at the rhythm and tempo levels. This mechanism enables the model to focus more on key features related to atrial fibrillation detection within the ECG signals, thereby improving the accuracy of predictions. From a medical point of view, the diagnostic criteria of AF are mainly the changes in the morphology of the ECG signal caused by the inconsistent RR interval and the disappearance of the P wave. These two morphological changes have been taken into account in Section 2.5.3 and Section 2.5.4 of our models.

#### 2.5.2. Signal Preprocessing and Segmentation

Prior to atrial fibrillation recognition, it is indispensable to subject the single-lead ECG signal x to a meticulous series of preprocessing steps, aimed at ensuring high data quality and enhancing the accuracy of subsequent analyses. Initially, a bandpass filter (typically set within the range of 0.5 Hz to 40 Hz) is utilized to eliminate high-frequency noise and low-frequency drift in the signal, thereby ensuring its clarity. Herein, the cutoff frequency for the low-pass filter, $F_{LP}$, is set to 40 Hz, and that for the high-pass filter, $F_{HP}$, to 0.5 Hz. The selected bandpass filter is a finite impulse response (FIR) filter, the length and design of which are determined based on the sampling rate of the signal and the characteristics of the required frequency band. Subsequently, the signal is normalized to eliminate differences in amplitude, using the z-score normalization method.

After preprocessing, the ECG signal x is segmented into M equal-length segments, denoted as s. In contrast to previous deep learning models that employed QRS complex detection for segmentation, which is susceptible to signal quality issues, we opt not to use it. Instead, a straightforward sliding window method is employed for segmentation. By setting the index range for each *i*th segment from $\left(\left(i-1\right)\times T\right)$ to $\left(\left(i\times T\right)-1\right)$, M segments of equal length T are obtained. This method enables more detailed analysis and modeling of each segment.

#### 2.5.3. Heartbeat-Level Attention Convolutional Layer

The core of the attention mechanism is defined by the formula:

(8) $$\mathrm{Attention}\left(Q,K,V\right)=\mathrm{Softmax}\left(\frac{QK^{T}}{\sqrt{d_{k}}}\right)V$$

where $d_{k}$ represents the dimensionality of the keys (key). This formula begins by calculating the dot product similarity between the queries (query, Q) and keys (key, K), followed by normalization through the Softmax function, and finally multiplying by the values (value, V) to produce a weighted output. Q, K, and V are obtained by linearly transforming the input matrix X with different trainable parameter matrices $W^{Q},$ $W^{K}$, and $W^{V},$ respectively:

(9) $$Q=XW^{Q}$$

(10) $$K=XW^{K}$$

(11) $$V=XW^{V}$$

where $W^{Q}$, $W^{K}$, and $W^{V}$ are trainable parameter matrices. The input matrix X is linearly transformed by multiplying with these three parameter matrices to generate the matrices Q, K, and V.

To effectively capture the beat-level features in ECG signals, especially for recognizing abnormal waveforms or edge features, we designed a heartbeat-based attention convolutional layer. This layer, leveraging the local connections, weight sharing, and pooling operations of convolutional neural networks, efficiently processes abnormal waveforms and edges in the signals, demonstrating outstanding performance. Notably, we introduce attention mechanism parameters $W^{Q}$, $W^{K}$, and $W^{V}$, using features extracted by the convolutional layer to guide the attention and improve interpretability at the beat level.

The process unfolds as follows: The signal undergoes a differentiation operation $\left(\mathrm{diff}\left(x\right)\right)$, followed by a padding function $\left(\mathrm{pad}\left(x\right)\right)$ to compensate for dimension changes caused by differentiation, highlighting the rate of change in the signal. The differentiated and padded signal is then processed through a one-dimensional convolution operation $\left(Conv\left(x\right)\right)$, with different convolutional kernels generating specific convolutional features for Q, K, and V. These convolutional features are normalized $\left(\mathrm{norm}\left(x\right)\right)$, distributing weights between 0 and 1, which helps stabilize computation and gradients. Finally, the normalized convolutional features are used in a matrix multiplication operation with the original signal x to generate $Q_{heartbeat}$, $K_{heartbeat},$ and $V_{heartbeat}$.

(12) $$Q_{heartbeat}=x\cdot {norm\left({Conv}_{Q}\left(pad(diff(x)\right)\right)}^{T}$$

(13) $$K_{heartbeat}=x\cdot {norm\left({Conv}_{K}\left(pad(diff(x)\right)\right)}^{T}$$

(14) $$V_{heartbeat}=x\cdot {norm\left({Conv}_{V}\left(pad(diff(x)\right)\right)}^{T}$$

Through this methodology, our model focuses more precisely on key locations within the ECG signal, enhancing its ability to recognize and analyze beat-level features and providing robust support for accurate anomaly detection in heartbeats. The design of this layer not only improves the model’s interpretability regarding heartbeat signals but also enhances the overall performance and interpretability of the model. The CNN in the heartbeat-level attention convolutional layer is instrumental in discerning intricate morphological details. This layer is trained to identify small fluctuations in the data by exploiting the property that the convolution operation is sensitive to data fluctuations. By guiding the attention mechanism with features by convolution, we hope that these features encapsulate the subtle differences in P-wave patterns in various signals as much as possible, thus enhancing the model to distinguish AF based on these subtle morphological changes to some extent. 

#### 2.5.4. Rhythm-Level Attention Recurrent Layer

For the identification of rhythm-level patterns, our focus lies in detecting abnormal rhythm variations within the ECG signal, crucial for accurately capturing arrhythmic events. The gated recurrent unit (GRU) [53,54], known for its sensitivity to time series data, serves as our ideal tool for analyzing rhythm changes. Incorporating a knowledge-guided attention mechanism allows us to effectively integrate physiological knowledge at the rhythm level, enhancing the model’s focus on rhythm-level concerns and its ability to recognize and interpret significant rhythm changes within the signal. The implementation is as follows:

For a given ECG signal $x\in R^{N\times T}$, where N represents the batch size and T the sequence length, we first apply a differentiation operation to highlight signal changes, followed by padding to maintain sequence length:

(15) $$\mathrm{Diff}\left(x\right)=\left[x\left[:,0,:\right],x\left[:,1:,:\right]-x\left[:,:-1,:\right]\right]$$

The signal, after differentiation and padding, is processed by a GRU network. This network is preferred over traditional LSTM networks due to its simplified structure, which improves computational efficiency while preserving the ability to capture long-term dependencies. Features necessary for query, key, and value are extracted using three distinct configurations of the GRU network:

$${\mathrm{GRU}}_{Q}\left(\mathrm{Diff}\left(x\right)\right),{\mathrm{GRU}}_{K}\left(\mathrm{Diff}\left(x\right)\right),{\mathrm{GRU}}_{V}\left(\mathrm{Diff}\left(x\right)\right)$$

The GRU outputs are transformed into corresponding weights through linear transformation layers, followed by normalization to ensure weight distribution between 0 and 1, enhancing model learning efficiency:

(16) $${W_{Q}}_{rhythm}=\mathrm{softmax}\left({\mathrm{Linear}}_{Q}\left({\mathrm{GRU}}_{Q}\left(\mathrm{Diff}\left(x\right)\right)\right)\right)$$

(17) $${W_{K}}_{rhythm}=\mathrm{softmax}\left({\mathrm{Linear}}_{K}\left({\mathrm{GRU}}_{K}\left(\mathrm{Diff}\left(x\right)\right)\right)\right)$$

(18) $${W_{V}}_{rhythm}=\mathrm{softmax}\left({\mathrm{Linear}}_{V}\left({\mathrm{GRU}}_{V}\left(\mathrm{Diff}\left(x\right)\right)\right)\right)$$

Finally, by performing dot product operations between the processed signal x and the normalized weights, query, key, and value are generated, completing the attention mechanism construction:

(19) $$Q_{rhythm}=x\cdot {{W_{Q}}_{rhythm}}^{T},K_{rhythm}=x\cdot {{W_{K}}_{rhythm}}^{T},V_{rhythm}=x\cdot {{W_{V}}_{rhythm}}^{T}$$

Through these steps, our model not only captures significant rhythm changes in the ECG signal but also effectively enhances the accuracy and interpretability of arrhythmia detection through the knowledge-guided attention mechanism. This process underscores the potential and advantages of deep learning models in ECG signal processing, particularly in rhythm-level analysis. The rhythm-level attention recurrent layer utilizes the power of GRU, a variant of recurrent neural networks, to analyze and interpret the sequential patterns of ECG rhythms. GRU is good at capturing temporal dependencies, making it partly an ideal tool for identifying abnormal RR intervals, a hallmark of atrial fibrillation. After training, this layer operates on higher feature dimensions to guide the attention mechanism to extract features. Somewhat further enhances the ability of the model to distinguish between normal and abnormal rhythms based on the inherently complex, time-varying features of AF signals.

#### 2.5.5. Fusion and Prediction

In our deep learning analysis of ECG signals, we employ an innovative end-to-end architecture integrating two key attention modules: a beat-level recurrent attention layer and a heartbeat-based attention convolutional layer. Furthermore, the model dynamically adjusts its focus on different parts of the ECG signal through a fully connected layer, optimizing its ability to predict atrial fibrillation.

Adaptive allocation of attention weights, guided by knowledge, fuses the beat-level recurrent attention layer with the heartbeat-based attention convolutional layer. Given the heartbeat-level features ${Attention}_{heartbeat}$ and rhythm-level features ${Attention}_{rhythm}$, the model computes the contribution of each feature type to the final prediction and dynamically adjusts their weights. Combining both beat-level and heartbeat-level features, along with their weights, the model generates the final prediction probability $P_{AF}$, determining whether the ECG signal indicates the presence of atrial fibrillation:

(20) $${Attention}_{heartbeat}=\mathrm{Softmax}\left(\frac{Q_{heartbeat}\times {K_{heartbeat}}^{T}}{\sqrt{d_{k}}}\right)V_{heartbeat}$$

(21) $${Attention}_{rhythm}=\mathrm{Softmax}\left(\frac{Q_{rhythm}\times {K_{rhythm}}^{T}}{\sqrt{d_{k}}}\right)V_{rhythm}$$

(22) $$P_{AF}=\mathrm{sigmoid}\left(\mathrm{softmax}\left(\mathrm{FC}\left(\mathrm{concat}\left({Attention}_{heartbeat},{Attention}_{rhythm}\right)\right)\right)\right)$$

where the (sigmoid) function maps the output to a probability space between 0 and 1, representing the likelihood of the signal being atrial fibrillation. (FC) denotes a fully connected layer, and (concat) indicates the operation of feature concatenation.

## 3. Experimental Methodology and Results

The post-training model undertakes AF identification upon receiving ECG signals. The experiments were conducted on a workstation equipped with an Intel i7 9700k CPU, NVIDIA GeForce RTX 4090 GPU, and 24 GB RAM, leveraging the PyTorch framework. The dataset, divided into records, was organized into a training set (75%), validation set (10%), and test set (15%), constituting the foundation for all training and evaluation tasks. Model optimization was facilitated by the Adam optimizer, initialized with a learning rate of 0.001, halved every 10 epochs in the absence of improvement on the validation set. A batch size of 96 was employed to optimize computational resources and learning stability, with cross-entropy loss utilized for this classification task. Performance metrics comprised accuracy, precision, recall, and F1-score. 

In Section 2.5.2, preprocessing and segmentation of single-lead ECGs, denoted as x, sampled at 300 Hz and with a duration of 9000 samples, were carried out. Segments of length T = 3000 samples were extracted with a step size of 500 samples, thereby dividing the ECG signal x into M = 12 equal-length segments. Section 2.5.3 details the heartbeat-level attention convolutional layer, configured with a depth of 2. This layer employs 8 attention heads, each with a dimensionality of 8, balancing expressiveness and computational efficiency. Three distinct one-dimensional CNNs, each configured with 32 filters, a kernel size of 3, a stride of 1, and padding of 1, guide the initialization of query (Q), key (K), and value (V) for the attention mechanism. Section 2.5.4 discusses the beat-level attention recurrent layer, also featuring a depth of 2, and maintaining the configuration of 8 attention heads with a dimension size of 8. For initializing QKV, three separate GRU (gated recurrent unit) layers are adopted, each GRU composed of 2 layers with 32 hidden units. Subsequently, a linear layer follows each GRU, transforming the segment length input of 3000 to an output dimension of 64, aligning with the combined head count and dimension size for attention computations. 

**Figure 7:**
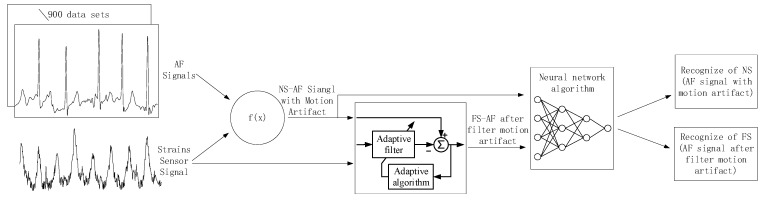
Flowchart of experimental procedure.

Figure 7 summarizes the experimental procedure. In the experiment, (a) we extracted 771 AF recordings from the MIT-BIH Database (MIT-BIH contains a total of 8528 ECG recordings) to serve as the original dataset without added MA interference. (b) Subsequently, we obtained the deformation of the skin surface on the right chest during running exercise from a stretch sensor. (c) Referring to the Equation (1), we superimposed the stretch sensor signal onto the AF signal to generate an artificially synthesized AF signal with MAs, termed the noise signal (NS). (d) We employed an adaptive filtering algorithm (described in Section 2.4) to process both the AF signal with MAs and the stretch sensor signal, resulting in an AF signal with MAs removed, termed the filter signal (FS). (e) Finally, we used a neural network recognition algorithm to process both the AF signal with MAs and the AF signal without MAs, obtaining recognition results for each signal.

In this experiment, a volunteer participated after being informed of the relevant experimental content. The volunteer wore a self-developed, single-lead ECG detection module [10] with ECG electrodes placed on the right chest and hips. Additionally, a stretch sensor was attached to the left and lower sides of the ECG electrode on the right chest, with a 0.5 cm interval. During the experiment, the volunteer sat in a chair for 10 min of rest before the experiment began. The experiment consisted of a 30-s period of standing still (a), followed by 30 s of running exercise, where arm swinging induced skin stretching on the chest (b). Afterward, there was another 10-s period of standing still (c). Each experiment lasted for 70 s, and a total of five experiments were conducted. Among these five experiments, the most stable result was selected for subsequent data analysis. Figure 7 presents the results of this selected data.

**Figure 8:**
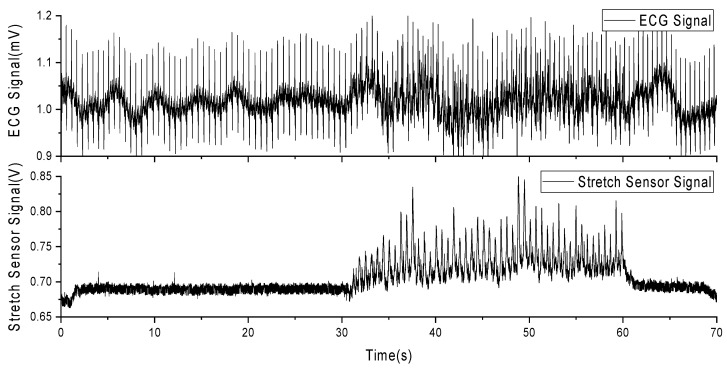
Experimental data of MAs from ECG and stretch sensors during running exercise.

As observed in Figure 8, (a) during the initial 30 s of stillness, the stretch sensor signal remains stable, and the ECG signal is relatively stable without significant interference. (b) From 30 to 60 s, during the running exercise, the stretch sensor detects a pronounced skin stretching signal, and there are evident MAs present in the ECG signal. (c) In the final 10 s of stillness, the stretch sensor signal returns to a stable value, and the ECG signal also stabilizes. This indicates that skin stretching is a source of MAs in ECG signals and is correlated with these artifacts.

**Figure 9:**
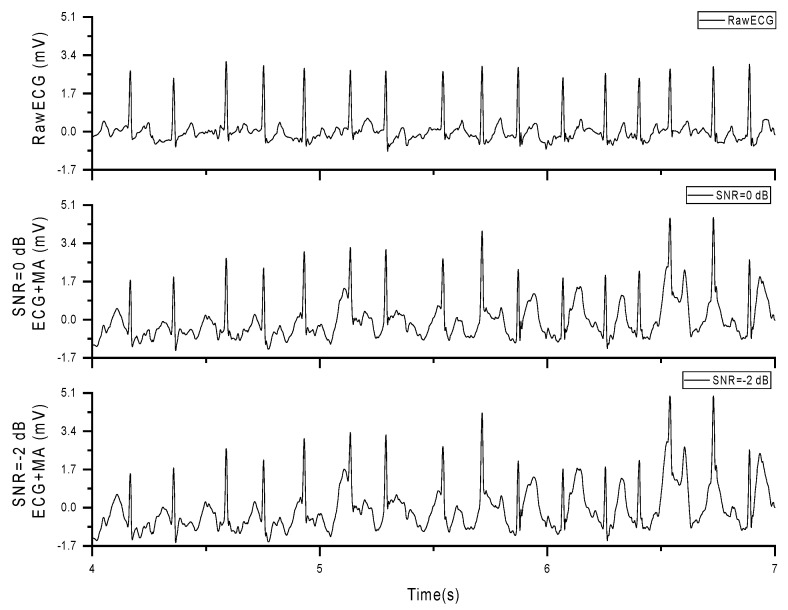
Original AF signal and AF signal with added MAs (SNR = 0 dB, −2 dB).

In subsequent experiments, we superimposed the stretch sensor signal onto the AF database using the formula presented in Section 2.3, resulting in AF signals containing MAs, referred to as noise signal (NS), as shown in Figure 9.

In the experiment, we modified the weighting coefficient ‘a’ in Equation (2) to obtain 13 different noise signals (NS) with a uniformly distributed SNR ranging from +8 to −16 dB. Subsequently, we analyzed the correlation between the NS signals and the stretch sensor signals to obtain correlation data under different SNR conditions, as presented in Table 3. As observed from Table 3, the correlation between the two signals increases as the SNR decreases, indicating an increase in the energy of MA.

The core of correlation analysis lies in computing the correlation coefficient between two signals. Among the most commonly used is the Pearson correlation coefficient, which quantifies the strength and direction of the linear relationship between two variables. The value of the correlation coefficient ranges from −1 to 1, where 1 indicates a perfect positive correlation, −1 indicates a perfect negative correlation, and 0 indicates no correlation.

In this formula, x and y represent the observed values of the signals, and n is the number of observations.

**Figure 10:**
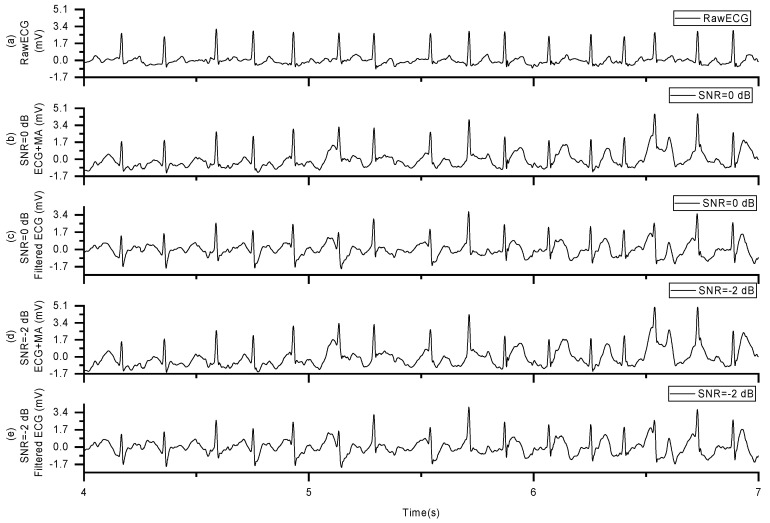
AF Signal with MAs removed: (**a**) the raw ECG data, (**b**) ECG plus MAs with SNR = 0 dB, (**c**) filtered ECG of signal in (**b**,**d**) ECG plus MAs with SNR = −2 dB, (**e**) filtered ECG of signal in (**d**).

Now, we have two sets of signals: one is the AF signal contaminated with MAs, and the other is the stretch sensor signal. Subsequently, we fed these two signal sets into an adaptive filtering algorithm. After processing with the adaptive filtering algorithm, we obtained the AF signal with MAs removed, referred to as the filter signal (FS), as shown in Figure 10.

The formula for calculating the Pearson correlation coefficient is given as follows:

(23) $$\left[r=\frac{n\left(\sum xy\right)-\left(\sum x\right)\left(\sum y\right)}{\sqrt{\left[n\sum x^{2}-{\left(\sum x\right)}^{2}\right]\left[n\sum y^{2}-{\left(\sum y\right)}^{2}\right]}}\right]$$

We further examined the signal-to-noise ratio (SNR) of the filter signal (FS) after removing MAs from 900 sets of noise signals (NS) with different SNRs to assess the effectiveness of motion artifact removal, as presented in Table 4. Our findings indicate that as the SNR of the NS decreases, the SNR of the FS after adaptive filtering to remove MAs also decreases. This suggests that while the adaptive filtering can eliminate a portion of the MAs, it does not completely remove all the motion artifact energy.

The basic formula for calculating SNR is shown in Equation (1). The formula for computing the SNR of the FS after removing MAs from the NS is given as follows:

(24) $$\mathrm{SNR}\;(\mathrm{dB})=10\cdot \log_{10}\left(\frac{P_{{signal}_{NS}}}{P_{{noise}_{NS}}}\right)$$

In this formula, ${signal}_{NS}$ represents the signal after removing MA from the NS, ${noise}_{NS}$ =${\;signal}_{NS}\;$− ${signal}_{origin}$, where ${signal}_{origin}$ is the original signal without added noise. P refers to the power of the signal.

Neural network algorithm described in Section 2.5 is to identify both the NS and FS, resulting in the detection of AF. By comparing our findings with the annotations provided in the MIT-BIH database, we obtained the final accuracy data for AF detection.

**Figure 11:**
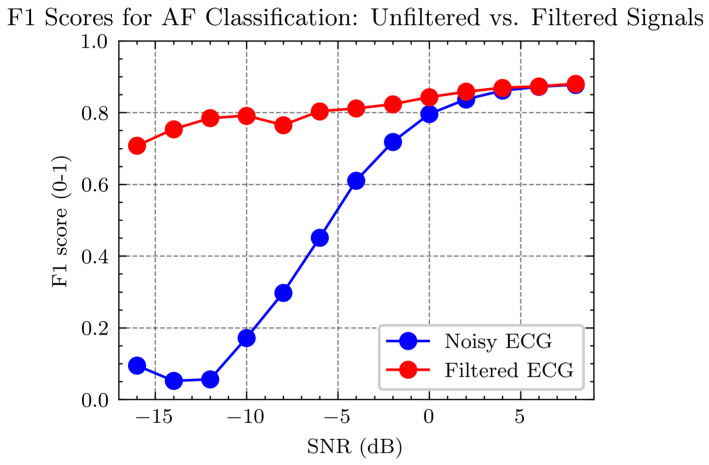
Comparison of F1 Scores for AF detection in noisy vs. filtered ECG signals at different SNR levels.

Figure 11 shows the F1 scores for detecting ECG signals at varying SNR levels. The F1 score is a metric that measures the balance between the precision and recall of a classification model, particularly in datasets where class distribution is imbalanced. It is the harmonic mean of precision and recall, thus requiring both to be high for the F1 score to be high. In binary classification tasks, a high F1 score indicates that the model is both accurate and comprehensive in identifying positive cases (such as positive disease diagnoses). The F1 score is especially crucial for applications where the cost of both false negatives and false positives is significant.

In Figure 11, two curves are presented: the red curve represents the F1 score for AF detection in noisy ECG signals—those not subjected to any filtering process. The blue curve signifies the F1 score for AF detection in filtered ECG signals—those processed to remove noise using a filter. The graph illustrates that the F1 scores for AF detection improve with an increase in SNR for both conditions, but the filtered ECG consistently demonstrates higher F1 scores across the SNR spectrum than the noisy ECG. Particularly at low SNR levels (SNR < 0 dB), filtering significantly enhances the F1 score for AF detection, underscoring the effectiveness of filtering in improving AF detection performance in low-quality ECG signals.

## 4. Discussion: Accuracy of AF Detection

After performing the aforementioned experiments on 900 sets of AF data, we obtained the AF detection accuracy with and without MAs, as shown in Figure 10. The red dots represent the AF detection accuracy with MA across a signal-to-noise ratio (SNR) range of −16 to 8, while the blue dots represent the accuracy with MAs removed within the same SNR range. From the figure, it can be observed that when the SNR is above four, the AF detection accuracy is nearly identical for both signals with and without MAs. However, when the SNR is below two, the AF detection accuracy for signals containing MAs drops significantly, nearing 0%. In contrast, the accuracy for signals with MA removed does not decrease as sharply, slowly declining to around 70%.

The accuracy in Figure 10 is calculated by the following equation:

(25) $$F1\;Score=2\times \frac{Precision\times Recall}{Precision+Recall}$$

where recall and precision are obtained using the following formulas:

(26) $$Recall=\frac{TPR}{TPR+FNR}$$

(27) $$Precision=\frac{TPR}{TPR+FPR}\;$$

**Figure 12:**
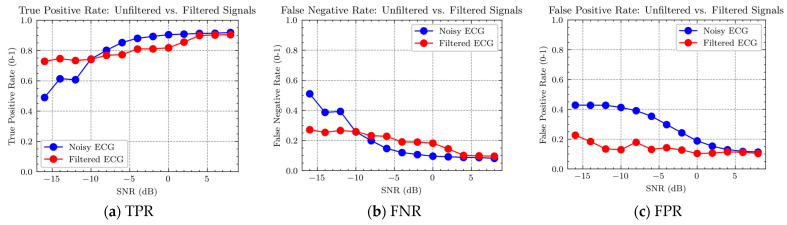
Ratio of true positive rate (TPR), false negative rate (FNR), and false positive rate (FPR) for AF detection in AF signals with and without MAs.

Corresponding to the three parameters in the above equation, the results obtained in the experiment are shown in Figure 12.

From Figure 12a,b, it can be observed that for AF signals containing MAs, there are fewer true positive detections and more false negative detections when the signal-to-noise ratio (SNR) is below −12 dB. However, when the SNR is above −10 and less than 4, there are more true positive detections and fewer false negative detections.

These results suggest that when MAs are strong, it is necessary to remove them for accurate AF detection. However, when MAs are not strong, the algorithm for removing them may introduce additional distortion, leading to poorer detection results. Therefore, the algorithm can first identify the strength of MAs and then apply different data processing strategies for different levels of MAs.

It is evident from Figure 12a,b that the separation between the red and blue points is smaller when the SNR is above −10 dB and less than 4 compared to when the SNR is below −12. Statistical analysis of this observation is presented in Table 5. It can be seen that the difference between the red and blue points for strong MAs with an SNR below −10 dB is three times greater than that for weak MA with an SNR above −10 dB and less than 4 dB.

The results suggest that the distortion caused by the motion artifact removal algorithm leads to a decrease in AF detection accuracy in the presence of low-intensity MAs, although the magnitude of this decrease is smaller than the improvement achieved in high-intensity MAs. In other words, if the strength of the MA is not distinguished and the algorithm is applied to all intensities of MAs, the improvement in detection accuracy achieved in high-intensity MAs is significantly higher than the decrease in detection accuracy achieved in low-intensity MAs.

From Figure 12c, it can also be observed that AF signals containing MAs exhibit fewer false positive detections compared to signals with MAs removed. This finding demonstrates the significant role of motion artifact removal methods.

## 5. Conclusions

The aim of this study was to investigate the accuracy of AF detection in signals with artificially added MAs after adaptive filtering. To achieve this, we used the AF dataset from the PhysioNet Challenge 2017 database and artificially added motion artifact signals with SNR ranging from 8 dB to −16 dB. First, we performed AF detection using an artificial intelligence algorithm on these signals with MAs to obtain the detection accuracy in the presence of MAs. This step provided baseline data for subsequent comparative analysis. Next, we applied adaptive filtering to these signals to remove the MAs. After adaptive filtering, we performed AF detection again to obtain the detection accuracy without MAs. By comparing the AF detection accuracy with and without MAs, we could assess the impact of adaptive filtering on AF detection. We found that in the case of strong MAs, the motion artifact removal algorithm improved the accuracy of AF detection, while in the case of weak MAs, the algorithm slightly decreased the accuracy. We also found that the AF detection accuracy of the signals after adaptive filtering improved across all intensities of MAs, with a maximum improvement of 60%. Our study demonstrates that, given a reference signal strongly correlated with MAs, the use of adaptive filtering techniques can significantly improve the accuracy of AF detection in ECG signals with strong MAs.

Stretch deformation, being one typical source of MAs within ECG recordings, is primarily studied in this paper. As running is the most common cause of MAs, our research mainly focuses on MAs induced by running. Additionally, other activities or body movements, including respiratory and muscular activities, could also induce MAs in the ECG signal. Due to the complexities of detecting these activities using conventional sensors, they pose great challenges in signal interpretation. Our future research will explore advanced methodologies to more accurately detect and analyze these diverse sources of artifacts, ranging from subtle movements between the sensor and the body to broad types of physical activity.

## Figures and Tables

**Table 1:** Characteristics of motion artifact signal.

	Amplitude	Frequency	Source of the MA
MA	Maximum 500% ECG peak amplitude	1–10 Hz	Changes in charge distribution at the interface between skin and electrodes

**Table 2:** The type of reference signal sensor and its corresponding research literature.

Type of Reference Signal Sensor	References
Impedance of electrode skin interface	[10,23,24,25,26,27,28,29,30]
Skin deformation	[23,31,32,33]
Triaxial acceleration	[25,26,33,34,35,36,37,38,39,40,41,42,43]
Others	[13,44,45,46,47,48,49]

**Table 3:** Correlation between NS signals and stretch sensor signals under different SNR Conditions.

SNR (dB)	Correlation of NS and Stretch Sensor Signal	SNR (dB)	Correlation of NS and Stretch Sensor Signal
8	0.373	−6	0.894
6	0.45	−8	0.929
4	0.536	−10	0.954
2	0.624	−12	0.969
0	0.708	−14	0.981
−2	0.784	−16	0.988
−4	0.846		

**Table 4:** Average SNR of the FS after removing MAs from 900 sets of NS with different SNRs.

SNR (dB)	Average SNR (dB)	SNR (dB)	Average SNR (dB)
8	6.314	−6	−1.239
6	5.625	−8	−1.277
4	4.828	−10	−3.66
2	1.767	−12	−7.324
0	0.292	−14	−9.063
−2	0.188	−16	−9.883
−4	−0.053		

**Table 5:** Classification of the gap between red and blue points based on true positive and false negative for different signal-to-noise ratio ranges.

Gap between Red and Blue Points	True Positive	False Negative
−10 < SNR (dB) < 4	0.053	−0.053
SNR (dB) < −10	−0.166	0.166

## Data Availability

Data are contained within the article.
